# IUI needs fairer appraisal to improve patient and stakeholder choices

**DOI:** 10.5935/1518-0557.20200066

**Published:** 2021

**Authors:** Gulam Bahadur, Bryan Woodward, Megan Carr, Santanu Acharya, Asif Muneer, Roy Homburg

**Affiliations:** 1 Reproductive Medicine Unit, North Middlesex University Hospital, Old Admin Block, London N18 1QX, UK; 2 X&Y Fertility, Leicester, UK; 3 Chelsea Westminster Hospital/West Middlesex Hospital University Trust, Twickenham Road, Isleworth TW7 6AF; 4 Ayrshire Fertility Unit, University Hospital Cross house, Kilmarnock, KA2 0BE, Scotland; 5 University College London Hospital, 250 Euston Road. London NW1 2PG; 6 Homerton Fertility Centre, Homerton University Hospital, London E9 6SR, UK

**Keywords:** IVF, IUI, cost efficiency, public health, stakeholder

## Abstract

Information supporting IVF at the expense of intrauterine insemination (IUI) has become commonplace, but it lacks critical analyses. Data from poorly practiced IUI, without an equivalent comparison to IVF, has been generalised to recommend a total abandonment of IUI in favour of IVF treatment. Our intention with this paper is to reappraise and balance arguments so that patients and stakeholders can have an unbiased informed choice. We provide information that reveals IUI to predominate over IVF in terms of integrated success, risks and cost to deliver one live birth whilst obviating the maternal and neonatal costs. Exceptional cost savings are demonstrated for IUI over IVF for fee-paying agencies and patients with lowered risks of maternal and neonatal care along with other risks including OHSS, fetal reduction and termination of pregnancies. This analysis supports the view that patients and stakeholders can choose IUI instead of IVF in most instances, except with bilateral tubal blockage and severe male factor infertility. It is apparent that fertility clinics need to re-evaluate and reconsider this field, and IUI can be of benefit to both subfertile patients and the stakeholders.

## BACKGROUND

Questions on how RCTs and the potential selection bias compared to big data, are particularly relevant for ART, where there is interest. Crucially, the UK’s National Institute of Health and Care Excellence (NICE) recommended that IUI treatment should be replaced by 3 cycles of IVF treatment, following appraisal of IUI studies with very low doses of clomiphene citrate (CC) (25mg) and without comparative data (Bahadur *et al*., 2017; Wordsworth *et al*., 2011). The FASST (Fast Track and Standard Treatment) trial had several weaknesses due to the in-built biases omitting IUI/hMG cycles and suggesting the premature use of IVF (Reindollar *et al*., 2010). That IUI/hMG serves a potential ‘threat’ to IVF is encapsulated in a further meta-analysis that focussed solely on high risk IUI/hMG studies, and then concluded that IUI/hMG should not be practiced whatsoever (Hansen, 2020; Zolton *et al*., 2020). In our opinion, almost all of the cases in this meta-analysis would have warranted cancellation, and the paper (Hansen, 2020; Zolton *et al*., 2020) is biased in favour of IVF treatment. Furthermore, none of the limitations was made clear to stakeholders and this requires analyses that are more critical. Financial analyses on cost effectiveness have so far been conducted crudely and seem overly concerned to portray IUI treatment as cost-inefficient. However, such analyses select poorly practised IUI cases, which are then utilized by Clinical Commissioning Groups (CCG) as evidence for NICE (2014). The most recent Cochrane analyses acknowledges that IUI in a stimulated cycle may result in a higher cumulative live birth rate compared to natural cycle IUI (Ayeleke *et al*., 2020).

With the proliferation of meta-analyses in the medical literature, these have come under considerable criticism for the level of arbitrary and selection biases (Page *et al*., 2014), raising questions regarding the validity of the data. Of particular interest is the systematic review and meta-analysis (Zolton *et al*., 2020) comparing live births and multiple gestations in couples with unexplained infertility undergoing IUI, following ovarian stimulation (OS-IUI) with oral medications versus gonadotropins. This study concludes that gonadotropin-stimulated IUI cycles in unexplained infertility could not be supported, and contrasts the largest comparative and integrated analyses between IVF and IUI (Bahadur *et al*., 2020). We express caution regarding the conclusions, which appear to preclude less invasive fertility treatments than IVF (Bahadur *et al*., 2020).

Interestingly, the eight studies chosen after rigorous selection could all be considered as ‘high risk’, since they include a decision to proceed to treatment, thus exposing mothers and babies to potential harm (Zolton *et al*., 2020). In two studies, no cancellation policy was presented, whilst the remaining studies permitted insemination with 3-7 follicles. Most practitioners would exercise caution with such high follicle numbers. Furthermore, non-cancellation might even be considered to amount to poor practice, unless there were a maximum of 3 follicles and where case-by-case assessment was made. There is no clear information as to how many mature follicles were present, which resulted in multiple births. The cases considered were not purely unexplained, and up to 50% of the cases appear to be mixed male factor; therefore, negating the authors’ claim that the strength of the study is in the number of 2,989 unexplained infertile couples (Zolton *et al*., 2020).

Whilst elective single embryo transfer (eSET) has been shown to be an effective strategy in reducing the number of multiple births after IVF cycles, it is inappropriate to compare this to high risk IUI practices if no comparative multiple birth data is presented in well managed IUI/hMG cycles. The authors allude to a cancellation of around 6.9% in their high risk IUI category, leaving the readers to imagine such a cancellation level would apply to all IUI gonadotropin well-managed cycles and that high multiple birth rates remains unavoidable.

More significant is the fact that the success rate for gonadotrophin-stimulated IUI cycles was 31.8%, which was much higher than the most recent IVF mean UK national figures from Human Fertilisation & Embryology Authority (HFEA), where even the best rates in women under the age of 35 years is 29% for IVF and 18% for OS-IUI (HFEA, 2020a; b). This point needs to be positively harnessed and worked in ways to minimise multiple births. IVF remains the single most important factor for multiple births and comparisons with high-risk IUI cycles serves an unnecessary distraction (Bahadur *et al*., 2020). Numerous well-constructed evidence-based studies support IUI (Bensdorp *et al*., 2015; Nandi *et al*., 2017; Tjon-Kon-Fat *et al*., 2017). The recent Cochrane review acknowledges IUI in a stimulated cycle may result in a higher cumulative live birth rate when compared to treatment with IUI in a natural cycle review (Ayeleke *et al*., 2020).

The USA does not classify IUI as an ART procedure. However, if it were included, this could potentially highlight to patients and funding agencies the option of a low risk, cost-effective treatment option. The largest integrated and comparative study undertaken on this topic places IUI in the context of IVF without the biases seen in numerous papers (Bahadur *et al*., 2020). This concludes that patients and stakeholders would well be advised to undergo IUI before IVF in most cases. The baseline IUI: IVF success rates to deliver a live birth (LB) was 2.35:1, which was much narrower than the RCT reported of 3:1. A small improvement in IUI LBR from 12.1% to 15.6% LBR narrows this difference to 1.73:1. The paper informs patients and stakeholders that 3.7 IVF cycles or 8.69 IUI cycles at 12.1% LBR or 6.4 cycle for a 15.6% LBR IUI are required to achieve a 100% theoretical LB. Despite creative ways of presenting IVF success rates, 70% of the women will never achieve an IVF baby. The multiple births for IVF were significantly greater than for IUI, despite the increasing eSET practice. IVF pregnancies were also associated with a 0.2% fetal reduction as a way to lessen multiple births. The paper reveals other risks for IVF, such as terminations for medical and a small number due to personal and social reasons. The knock-on effect for maternal and neonatal cost per year to the UK from IVF babies was £115 million, a cost burden not picked up by IVF clinics. The unique algorithms developed reveal that IUI clinics could deliver a cost-effective benefit per LB of £42 558, while extending this benefit to £76 257 for 15.6% LB IUI against IVF 27.3% LB. IVF clinics providing `add-on’ techniques simply increase the cost, thereby eroding any cost benefit IVF can deliver per LB, while increasing the cost efficiency of IUI LB. On an economic and scientific basis, it is worthwhile investing in IUI LB improvement, when considering non-evidenced based add-ons with no proven worth. By its own financial guidelines, NICE is compelled to inform UK CCGs to fund IUI before IVF, given the cost efficiencies deliverable from IUI procedures (Bahadur *et al*., 2020; NICE, 2014). For the first time this unfettered unique analysis provides fee-paying stakeholders, patients and governments crafting policies detailed information to make informed choices away from IVF clinics.

To conclude, the politics of influencing crucial bodies to construct treatment policies and funding criteria require interest groups to provide evidence through peer reviewed papers. Such bodies dismiss large grey data unpublished in peer reviewed journals. It has been all too easy to exploit these loopholes for those motivated to generate evidence on a variety of levels and in favour of more profitable IVF treatments despite weak to very weak evidence. It is therefore imperative to gain a balanced view of the field of ART dealing with highly sensitive and vulnerable patients. For the first time, a sizable and a bird’s eye view analyses make a compelling case for IUI treatment before embarking on IVF treatment, based on outcomes, risks and cost effectiveness. We recommend that fertility treatment policies are constructed to use the new information away from IVF clinic pressures.
